# Operating Theatre Readiness for Urological Emergencies: Challenges, Pitfalls, and a Practical Checklist for Urology On-Call Teams

**DOI:** 10.7759/cureus.97399

**Published:** 2025-11-21

**Authors:** Stefanos Gkaliamoutsas, John Gibson, Vaibhav Modgil, Ian Pearce, Theodora Stasinou

**Affiliations:** 1 Department of Urology, Manchester University NHS Foundation Trust, Manchester, GBR; 2 Faculty of Biology, Medicine, and Health, The University of Manchester, Manchester, GBR

**Keywords:** checklist, emergency surgery, human factors, quality improvement, theatre preparedness, urology

## Abstract

Urological emergencies demand prompt, skilled intervention to avoid serious complications. However, when these emergencies arise unexpectedly during non-urological procedures, such as general surgical, obstetric, gynaecological, or emergency operations, delays caused by inadequate equipment availability, lack of staff familiarity, poor communication, and other human factors can compromise patient outcomes. Drawing from a real case of intra-operative bladder and ureteric injury during an open inguinal hernia repair, this technical report highlights recurrent system barriers to timely emergency urological care and outlines practical solutions. A structured checklist is proposed to support rapid preparedness in non-specialist theatres, improve team coordination, and reduce avoidable delays. While descriptive rather than data-driven, the report synthesises real-world challenges and offers an adaptable, practice-oriented framework that may strengthen preparedness for high-risk urological emergencies.

## Introduction

Urological injuries sustained during non-urological operations, most commonly during obstetric, gynaecological, and colorectal procedures, present unique and underestimated risks. Procedures such as caesarean sections, hysterectomies, and colorectal resections carry a recognised risk of urological injury, notably to the bladder and ureters [1,2]. Reported rates of urinary tract injury during non-urological procedures range from 0.1% to 4%, depending on procedure type, with a higher incidence in complex gynaecological and colorectal operations [2-4]. These events are a major cause of delayed post-operative morbidity and commonly require urgent urological intervention [3]. However, managing such events in theatres that are not routinely equipped for urology creates practical challenges [4]. Urologists may be called into operating rooms with unfamiliar teams, limited resources, and limited access to essential diagnostics like cystoscopy or fluoroscopy [4].

The existing literature has historically focused on the technical aspects of repair rather than the team, equipment, and organisational factors that influence these situations [4]. However, a comprehensive approach that incorporates clinical acumen and non-technical skills is essential [5]. The primary aim of this technical report is to provide a concise, practice-oriented checklist to assist urology on-call teams and non-specialist theatre staff when managing unexpected urological emergencies. The intended audience, therefore, includes clinicians and operational staff involved in acute surgical care, rather than a research-focused readership. Although not a hypothesis-driven scientific study, this work follows a quality-improvement approach that aligns with contemporary patient-safety science.

## Technical report

Case illustration: when unpreparedness endangers outcomes

During an elective open inguinal hernia repair in a male patient, a suspected bladder and ureteric injury was identified. The urology registrar on-call was urgently requested. Upon arrival, it was evident that the operating theatre was inadequately prepared for managing such a complication. The cystoscopy stack was not present, and the theatre staff were unaware of the storage locations for guidewires and ureteric catheters. Significant time was required to locate the necessary equipment, arrange for a mobile image intensifier (e.g., C-arm), and request the presence of a radiographer.

The delay of approximately 90 minutes postponed the initiation of cystoscopy and ureteric stenting. There was no ensuing team debrief, and the urology team continued with their on-call duties for the remainder of the shift. This incident underscored the need for anticipatory planning and the establishment of clear, standardised protocols. Although intra-operative urological injuries are relatively uncommon, their consequences can be significant when they do occur. Appropriate preparedness is therefore essential to ensure timely and effective intervention, as well as appropriate debrief and support signposting for the involved healthcare professionals.

Common challenges identified

Several recurring issues complicate emergency care in urology theatres at National Health Service (NHS) hospitals. One major concern is equipment availability. Many general theatres lack urology-specific instruments such as cystoscopes, ureteric catheters, and guidewires as part of their standard inventory [4]. Even when these tools are present, they may be scattered across different storage areas or poorly maintained, leading to delays in setup and treatment. The absence of a dedicated and well-organised urology equipment stock significantly hinders the efficiency of emergency procedures.

Another key issue involves staff training deficits. Operating theatre nurses and radiographers outside dedicated urology teams often lack routine training in specialist urology instruments and emergency protocols, which may lead to delays or difficulties during theatre setup. Evidence from studies of operating-room staff training suggests that needs-based, continuous education is often under-utilised or delivered inconsistently, with targeted interventions shown to improve preparedness [6]. Similarly, urology workforce data indicate significant sub-specialisation and variation in consultant distribution and procedural exposure, suggesting that fewer surgeons may maintain broad competencies in open pelvic repairs or urgent ureteric injury management [7].

Access to imaging also poses substantial challenges. Fluoroscopy, or mobile image intensifier equipment, is frequently limited outside of regular working hours, and radiographer staffing levels may be reduced at night or on weekends. Even when fluoroscopy is available, many operating tables are incompatible with a C-arm, complicating the imaging process [4]. Transferring a patient, especially one with an open abdomen, onto a radiolucent operating table can be distressing and time-consuming, leading to further procedural delays.

Finally, communication and escalation failures contribute significantly to inefficiencies in emergency urological care. Delayed recognition of urinary tract injuries and late referrals to urology registrars or consultants can prolong theatre time and worsen patient outcomes [8,9]. In some hospitals, clear escalation pathways are lacking or inconsistently applied, resulting in uncertainty and delayed decision-making during critical situations. Collectively, these pitfalls can significantly delay definitive treatment, exposing patients to avoidable risks and potentially compromising their recovery [8,9]. To address these challenges, Table 1 outlines a checklist for emergency urology procedures created to support preparedness and streamline response during urgent cases. This checklist is intended for situations where a urological injury is suspected or confirmed and is not intended for routine use in all surgical cases. Additionally, it outlines minimum recommended actions and equipment; items are intended to be adapted to local staffing levels, resources, and theatre workflows rather than applied uniformly to every case.

**Table 1:** Emergency urology theatre readiness checklist A practical checklist designed to support rapid readiness and reduce delays during unexpected intra-operative urological emergencies in non-specialist theatres.

Section	Key Points
Pre-Operative Preparation	Review the surgical history and details of the suspected injury. Review the "five Ps": Patient, Position, Personnel, Planning, and Procedure [4]. Request and review imaging such as a CT scan of the kidneys, ureters and bladder. Escalate to the consultant urologist if appropriate, ensuring direct consultant-to-consultant discussion when required. Identify and confirm the location of essential equipment early.
Essential Equipment Checklist - Basic Urology Kit	Rigid cystoscope with sheaths (17–22 French gauge); light source, camera system, and stack; working bridge; irrigation tubing and saline; flexible cystoscope (ideal but optional); urethral dilators.
Essential Equipment Checklist - Ureteric Access Tools	Range of guidewires (hydrophilic and standard); 6Fr ureteric catheters; contrast mediums (Urografin or equivalent); access to a mobile image intensifier with sterile drapes; fluoroscopy-friendly (radiolucent) operating table with stirrups.
Essential Equipment Checklist - Intervention & Repair Kit	Semi-rigid ureteroscope; double-J ureteric stents (20–28 cm lengths); retrieval baskets or grasping forceps; ureteral access sheaths; absorbable sutures; suprapubic catheter set (e.g., Seldinger); Robinson drains; methylene blue; major pelvic surgical tray.
Team Briefing	Repeat introductions and confirm the aims of the urological intervention, including the likely course of action. Administer antibiotics covering gram-negative bacteria. Confirm the roles and responsibilities of all team members. Review the surgical plan and imaging requirements. Coordinate with anaesthetics and radiography teams.
Documentation and Post-Operative Care	Take intra-operative clinical photographs where appropriate and add them to the patient record. Complete a thorough operation note including intra-operative findings, instruments used, and any foreign bodies placed (e.g. ureteric stents, drains, and catheters). Outline the post-operative management plan with explicit instructions for non-urological staff caring for the patient. Conduct a team debrief and highlight available support for healthcare professionals.

## Discussion

Human factors and operating theatre culture

The role of human factors in urological emergency management has become increasingly recognised as integral to patient safety. As Brennan and Oeppen highlight, preventable harm frequently arises from communication breakdowns, unclear team roles, cognitive overload, and fatigue; all of these factors are often exacerbated during urgent requests for urological input in non-specialist theatres [10]. Urologists, when summoned to unfamiliar environments, may find themselves operating without key resources while also navigating high emotional tension among surgical staff. In such settings, human limitations must be anticipated and supported through systems that promote awareness, psychological safety, and clear leadership structures.

Delays similar to the 90-minute interruption described in our case have been noted anecdotally in other centres, where difficulties in locating cystoscopy equipment or guidewires or in obtaining fluoroscopy outside of hours are recognised contributors to prolonged time-to-intervention [4,11]. Although precise timings vary and formal quantitative audits are limited, these system constraints are widely acknowledged in patient-safety and emergency surgery literature as recurrent barriers to timely intra-operative urological care [9]. The challenges described in this technical report, therefore, reflect established themes rather than isolated observations.

Brennan and Oeppen also advocate for structured briefings and debriefings and the implementation of cognitive models such as HALT (Hungry, Angry, Late, Tired) to enhance situational awareness and reduce risk [10]. These frameworks become particularly relevant during out-of-hours emergencies or when teams are under pressure. In urology, the acute repair of iatrogenic injuries, especially to the bladder or ureters, requires not only technical precision but also calm, coordinated decision-making. A culture that encourages junior staff to speak up, reduces the authority gradient, and acknowledges emotional stress as a performance factor is essential for optimising outcomes in urological emergencies.

Urological competency gaps

At a broader level, urology has become increasingly sub-specialised, with almost 70% of UK consultants reporting at least one sub-specialty interest, according to the 2023 British Association of Urological Surgeons (BAUS) Workforce Report [7]. Although sub-specialisation provides clear benefits for planned elective care, it may also reduce the pool of consultants with regular exposure to low-volume open pelvic procedures that are occasionally required during acute repairs such as ureteric reimplantation. At the same time, consultant distribution varies markedly across the UK, with NHS urology departments ranging from 2.1 to 18.8 consultants per unit, further contributing to variability in the availability of suitable expertise during emergencies [7]. Together, these factors highlight the need for formal mechanisms to maintain competence in complex but infrequently performed procedures.

There may be merit in establishing a second-tier regional on-call rota with pelvic surgeon cover for complex cases, given the increasing sub-specialisation within urology and variability in consultant exposure to complex open pelvic procedures [7]. Complementary training initiatives, including cadaveric simulation, are recommended by the Royal College of Surgeons of England to maintain technical proficiency in low-frequency but high-stakes operations [12]. Reviewing the formal curriculum for urology specialist training may also be warranted to ensure that essential competencies for managing intra-operative urological injuries remain embedded within national training pathways [13].

Operative documentation and post-operative surveillance

In managing intra-operative urological injuries, meticulous documentation is essential. This includes detailed operative notes and, where possible, intra-operative photographs. Appropriate and timely post-operative follow-up is particularly important, as such injuries may have long-term implications for patients [1]. Daily reviews help detect evolving complications early, and any changes to the management plan should be clearly communicated to the parent team. Midwives and ward staff caring for catheters and drains must receive explicit post-operative instructions to avoid preventable errors, such as premature removal or incorrect handling.

Radiological investigations, including computed tomography (CT) cystography, retrograde studies, or renograms, should be arranged to document outcomes and monitor recovery [14]. Clear documentation also has significant medico-legal importance. Intra-operative urological injuries are high-risk events where failure to recognise, document, or appropriately manage the injury can result in substantial legal liability for both the surgeon and the institution [15]. Providing patients with clear, honest explanations about the nature of the injury, the intra-operative steps taken, and the planned follow-up is essential for both ethical and legal reasons.

Second victim syndrome and institutional responsibilities

The concept of the "second victim" recognises that healthcare professionals involved in adverse events often experience emotional harm [16]. These effects are particularly pronounced for clinicians confronted with high-stakes emergencies in unfamiliar or inadequately equipped environments. Effective institutional responses must go beyond reactive counselling and instead adopt structured, proactive approaches that normalise emotional support as part of routine patient-safety practice.

Such interventions might include peer-led debriefs following adverse events, access to multidisciplinary support services, and clear information regarding the potential psychological impact of such incidents [16-18]. Opportunities for reflective practice and structured mentoring can help clinicians process difficult experiences constructively. Organisational acknowledgement of the emotional labour inherent in surgical practice is vital, and staff wellbeing must be treated as an integral component of patient safety [17,18].

Professional bodies, including the NHS, as highlighted in the 2020 Patient Safety Incident Response Framework, advocate for support pathways that validate the emotional impact of adverse outcomes, reduce stigma, and equip clinicians with coping mechanisms [17]. Implementing formal "second victim" protocols improves not only individual well-being but also collective team resilience and organisational safety culture [18].

Practical applicability and adaptability to real-world settings

While the checklist proposed in this report incorporates learning from a real case of bladder and ureteric injury, it is not intended as a prescriptive standard for every hospital or every operation. Theatre resources, staffing models, and equipment availability vary widely between institutions. The checklist should therefore be viewed as a flexible framework that can be adapted to local needs, rather than an expectation that all items must be available at all times. Its purpose is to provide a structured readiness tool that supports safer, more coordinated responses to unexpected urological injuries in diverse clinical environments.

## Conclusions

Improving readiness for unexpected urological emergencies requires NHS trusts to prioritise cross-specialty induction for theatre staff, ensure access to essential equipment, strengthen escalation pathways, and embed routine debriefing and second-victim support structures. Readiness depends not only on the availability of tools but also on cohesive teamwork, clear leadership, and a culture that promotes communication and psychological safety. The checklist presented in this report is designed as a flexible, practice-oriented framework rather than a prescriptive standard. Because staffing levels, equipment availability, and workflow constraints vary widely across institutions, each organisation should adapt the recommendations to its own operational environment. The aim is to provide a structured baseline template that helps minimise avoidable delays and supports coordinated, confident responses to rare but high-risk intra-operative urological injuries.

Although this report is descriptive rather than data-driven, the recurring system issues highlighted mirror those documented in patient-safety literature. Future work could strengthen the evidence base by incorporating quantitative metrics such as time-to-intervention audits, equipment availability checks, incident-report analysis, and follow-up outcomes. Evaluating these measures over time would help determine the impact of readiness frameworks on patient care, clinician well-being, and organisational performance. Ultimately, integrating technical capability with a compassionate, well-supported team culture is essential for ensuring that surgical systems can deliver timely and effective care when unexpected urological emergencies arise. Embedding these principles into local policies and national guidance will be key to improving safety, resilience, and outcomes across surgical services.
